# 

**Published:** 1915-05-29

**Authors:** 


### CONTENTS.

PAGE

**The Hospital Needs of our British Forces** ... 177

**Hospitals and the Holiday Season** ... ... 178

**Hospital and Institutional News ...** ... ... 179

The New King George Hospital Opened—Hospitals and the Spirit Tax—The Fulham Military Hospital—Poor-Law Medical Officers and Certificates under the Mental Deficiency Act—The Cruciform Block—Deaths from Starvation—Dispensing in Mental Hospitals—Voluntary Aid in Mental Deficiency Work—Local Authorities and Voluntary Aid—The Secretaryship of Hampstead General Hospital—A "Sporting Spirit" in Religion—The Troop Train Disaster—A Farthing Rise in the Cost of Provisions—The Work of the Special Hospitals for Officers—Increasing the Staff at Hope Hospital—Women Doctors after the War—Hut-Wards in London—A New Medical Post—The Number of Doctors on War Service—Guardians and their Subscriptions to Hospitals—Extraordinary Expenditure in War-Time—The Hostel for Blinded Soldiers—British Pharmaceutical Conference—Increased Demand for Patent Medicines—Special Hospitals in Canada—This Week's Drug Market—To Our Readers.

**Discipline in the New Military Hospitals** ... 185

Problems Arising from an Initial Mistake.

**Traumatism and Disease** ... ... ... ... 186

Difficulties under the Workmen's Compensation Act.

**"The Teutonic Health Resorts"** ... ... ... 187

British Substitutes that Need Awakening.

**The Education of the Defective in Canada** ... 188

The Benefits of Auxiliary Classes.

[*See over.*]

### CONTENTS—(*continued*).

| | PAGE |
|---|---|
| **A Medical Causerie** ... ... ... ... ... | 189 |
| **Round the World** ... ... ... ... ... | 190 |
| Fellow-Workers and the Roll of Honour. | |
| **Hospital Architecture and Construction** ... | 191 |
| City Sanatorium, Birmingham. | |
| **Buildings Contemplated and in Progress** ... | 192 |
| **The Editor's Letter-Box** ... ... ... ... | 193 |
| Queen Mary's Convalescent Auxiliary Hospitals. | |
| The Annual Dinner and the War. | |
| **The Pharmaceutical Society and the War** ... | 193 |
| **The Department of Modern Nursing** ... ... | 194 |
| XIV.—Nurse Training in Three Cottage Hospitals: Teignmouth, Dawlish, Newton Abbot. | |
| **The Military Hospital, Sierra Leone** ... ... | 195 |
| By One Recently on the Staff. | |
| **The New Sanatorium at Leicester** ... ... | 196 |
| **Personalia** ... ... ... ... ... ... | vi |
| **Doctors' Wills** ... ... ... ... ... ... | vi |
| **Gifts and Bequests** ... ... ... ... ... | vi |
| **The Institutional Worker** ... ... (Suppl.) | 1 |
| Disinfection in Sanatoria: Answer to April Question. | |
| Question for May. | |
| Questions Invited. | |
| Index System for Minute-Books: Answer to April Question. | |
| Enquiries and Answers. | |
| Employment and Training: Hospital Laundry Work. | |
| Practical Matters: Simple and Effective Water Softener | |
| The Sick and in Need: Phthisical Child—After-Care. | |

